# MutY-Homolog (MYH) inhibition reduces pancreatic cancer cell growth and increases chemosensitivity

**DOI:** 10.18632/oncotarget.13985

**Published:** 2016-12-16

**Authors:** George Sharbeen, Janet Youkhana, Amanda Mawson, Joshua McCarroll, Andrea Nunez, Andrew Biankin, Amber Johns, David Goldstein, Phoebe Phillips

**Affiliations:** ^1^ Pancreatic Cancer Translational Research Group, Lowy Cancer Research Centre, Prince of Wales Clinical School, University of New South Wales, Sydney, Australia; ^2^ Children's Cancer Institute, Lowy Cancer Research Centre, University of New South Wales, Sydney, Australia; ^3^ Australian Centre for NanoMedicine, University of New South Wales, Sydney, Australia; ^4^ Wolfson Wohl Cancer Research Centre, Institute of Cancer Sciences, University of Glasgow, Bearsden, Glasgow, Scotland, United Kingdom; ^5^ The Kinghorn Cancer Centre, Cancer Program, Garvan Institute of Medical Research, Darlinghurst, Sydney, Australia

**Keywords:** pancreatic cancer, mutY-homolog (MYH), chemoresistance, oxidative stress, DNA repair

## Abstract

Patients with pancreatic ductal adenocarcinoma (PC) have a poor prognosis due to metastases and chemoresistance. PC is characterized by extensive fibrosis, which creates a hypoxic microenvironment, and leads to increased chemoresistance and intracellular oxidative stress. Thus, proteins that protect against oxidative stress are potential therapeutic targets for PC. A key protein that maintains genomic integrity against oxidative damage is MutY-Homolog (MYH). No prior studies have investigated the function of MYH in PC cells. Using siRNA, we showed that knockdown of MYH in PC cells 1) reduced PC cell proliferation and increased apoptosis; 2) further decreased PC cell growth in the presence of oxidative stress and chemotherapy agents (gemcitabine, paclitaxel and vincristine); 3) reduced PC cell metastatic potential; and 4) decreased PC tumor growth in a subcutaneous mouse model *in vivo*. The results from this study suggest MYH may be a novel therapeutic target for PC that could potentially improve patient outcome by reducing PC cell survival, increasing the efficacy of existing drugs and reducing metastatic spread.

## INTRODUCTION

Pancreatic ductal adenocarcinoma [herein referred to as pancreatic cancer (PC)] is a highly metastatic and chemoresistant disease with a dismal 5-year survival rate at 7% [1, 2]. Our current best treatment is surgery, but < 15% of patients present with resectable PC [3, 4]. For the remainder of patients, our current chemotherapeutic strategies only prolong median survival by 8-16 weeks [5]. Part of the reason PC treatments often fail, is the highly fibrotic PC microenvironment [6, 7]. Fibrosis distorts the tumor vasculature creating a hypoxic and nutrient-deprived environment [6, 7]. Hypoxia and nutrient-deprivation drive the Warburg effect (a switch from oxidative to glycolytic energy production) and the transition of cancer cells from an epithelial phenotype, to a more metastatic and chemoresistant mesenchymal phenotype [8–11]. These features also impose additional oxidative stress on PC cells. This is because hypoxia induces the release of reactive oxygen species (ROS) from mitochondria, initiating a signaling cascade to facilitate cell survival [12]. In addition, the Warburg effect results in loss of potent anti-oxidant intermediates, reducing the ability of cancer cells to mop-up ROS [9].

ROS are mutagenic as they are highly reactive with DNA bases. Intracellular ROS are generated by normal cell metabolism, but can be increased by microenvironmental pressures (as described above) and chemotherapeutics [8–11, 13]. If left ignored, DNA damage induced by oxidative stress can accumulate to a point that it induces cell death, either by inducing irreparable DNA double strand breaks or by accumulating excessive DNA mutation that compromises the basic functions of the cell [14]. Proteins involved in cellular responses that protect cells from oxidative stress are increasingly being recognized as potential therapeutic targets for cancer. For example, Kiebala et al. [15] demonstrated that dual inhibition of thioredoxin and glutathione antioxidant systems synergistically reduced malignant B cell viability but did not affect normal B cells, *in vitro*. Sulfasalazine is a drug that reduces glutathione production and cellular protection from oxidative stress, and has been shown to reduce pancreatic cancer cell viability [16]. MTH1 protects the genome from oxidative DNA damage by sanitising dNTP pools of oxidized DNA bases, preventing their incorporation into DNA [17, 18]. Inhibitors of MTH1 have been shown to be effective at killing cancer cells (including PC cells) *in vitro* and *in vivo* [17, 18]. The base excision repair (BER) pathway is a major player in cellular protection from oxidative stress and has been identified as a key regulator of resistance to a variety of chemotherapeutics [19, 20]. Targeting BER proteins involved in oxidative DNA damage repair may thus chemosensitize cancer cells and reduce their survival in a ROS-promoting microenvironment.

The most common form of oxidative DNA damage is the oxidation of guanines (G) to 8-oxo-guanine (8-oxo-G) in G:C base pairs. If the 8-oxo-G is not removed, replication machinery can mis-insert adenine (A) opposite 8-oxo-G, which becomes a permanent mutation (G:C to T:A) in subsequent rounds of replication [21]. The BER proteins OGG1 and MYH play major roles in repairing this damage. OGG1 directly removes 8-oxo-G, while MYH prevents this DNA damage from becoming a permanent mutation, by removing A incorrectly inserted opposite G [22]. MYH also plays a critical role in co-ordinating other BER proteins at these DNA damage sites including OGG1 to ensure repair is carried out correctly [23, 24]. MYH has been shown to interact with components of the mismatch repair (MMR) pathway, a DNA repair pathway that recognises DNA backbones deformities due to base mismatches [25]. Interactions with MMR proteins have been shown to enhance MYH activity rather than compete with it, indicating MYH plays a central role in repair of oxidative DNA damage [25].

Given the PC microenvironment promotes oxidative stress and that MYH plays an important role in protecting cells from oxidative DNA damage, we hypothesized that MYH may be a therapeutic target for PC. Despite its critical role in oxidative DNA damage repair, MYH has not been studied as a therapeutic target in any cancer. We show that MYH silencing using siRNA reduces PC cell survival and metastatic potential, and increases chemosensitivity *in vitro*. In addition, therapeutic inhibition of MYH using a nanoparticle siRNA approach significantly decreased PC tumor growth *in vivo*. The results from this work identify MYH as a novel therapeutic target for PC.

## RESULTS

### Expression of MYH in PC cells and knockdown of MYH using siRNA

**Figure 1:**
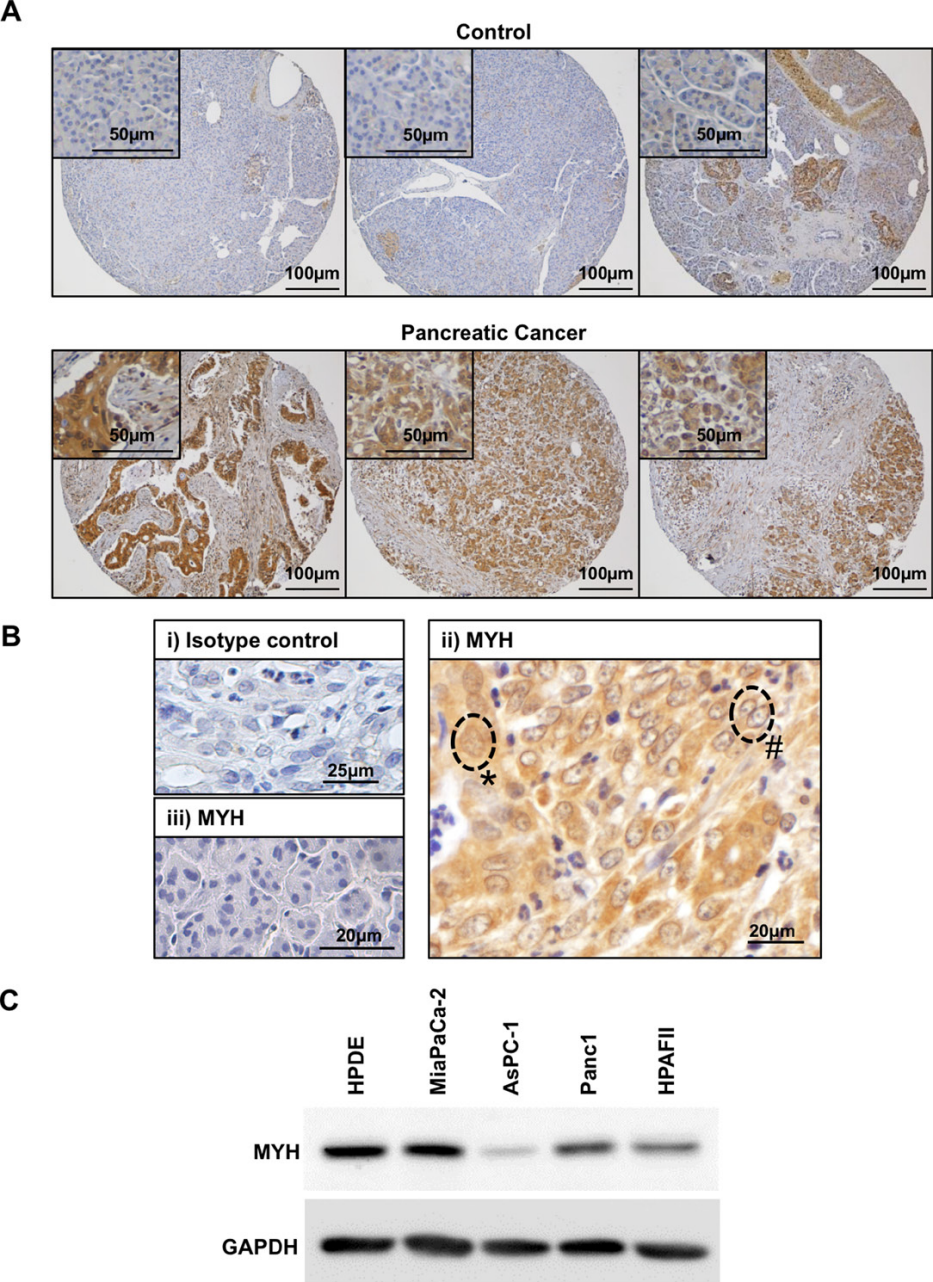
Expression of MYH in human pancreatic adenocarcinoma tissue and cell lines (**A**) Panels show control tissue (normal pancreas or benign pancreatic conditions) or pancreatic cancer tissue stained with MYH antibody (brown). Closer views of normal acinar cells (top panels) or pancreatic cancer cells (bottom panels) are shown in insets. (**B**) Magnified field of view showing human pancreatic cancer tissue stained with either isotype control antibody (i) or MYH antibody (ii-iii). The isotype control was negative and tumor elements had strong immunoreactivity for MYH. Labels demonstrate cytoplasmic+nuclear staining (*) versus cytoplasmic only staining (#). Panel (iii) demonstrates low MYH staining in normal acinar cells. (**C**) Western blot analysis of MYH in protein extracts from normal human pancreatic ductal epithelial cells (HPDE cells) and pancreatic cancer cell lines. GAPDH was used as a loading control.

To assess the expression of MYH in PC cells, we first analyzed MYH protein levels in human PC tissue specimens by immunohistochemistry. We observed abundant MYH in PC tumour tissue but not in control pancreatic tissue (normal pancreas or benign conditions; 16/20 pancreatic cancer tissue microarray specimens showed upregulation of MYH relative to the normal/benign pancreas cohort; Figure 1A). MYH was found to be highly expressed in PC cells (Figure 1Bii) with variable nuclear staining (Figure 1Bii). Variations in nuclear localization were most likely linked to cell cycle, as MYH localization has been shown to be primarily nuclear during S-phase and diffuse throughout other phases [26]. In contrast, MYH expression was low in normal acinar cells (enzyme-secreting cells of the pancreas) adjacent to the tumor (Figure 1Biii). We also investigated MYH expression in protein extracts from normal human pancreatic ductal epithelial cells (HPDE cells) and the PC cell lines MiaPaCa-2, HPAFII, Panc-1 and AsPC-1 (MiaPaCa-2, Panc-1: primary tumor-derived; HPAFII and AsPC-1: metastases-derived). Abundant MYH protein was detected in all lines including HPDE cells, though levels were variable between PC cells (Figure 1C). This variation was important as it represents the heterogenous nature of pancreatic cancer [27]. It should be noted that while primary HPDE cells represent “normal” pancreatic cells, these are cultured on plastic and are constantly proliferating unlike the situation *in vivo*. Nevertheless, it is not surprising that MYH would be expressed in non-cancerous cells, as it plays a role in protecting the mammalian genome against basal oxidative DNA damage (see introduction). We chose to pursue our experiments in MiaPaCa-2 and AsPC-1 cell lines as these represented primary and metastatic locations as well as varying expression of MYH.

**Figure 2:**
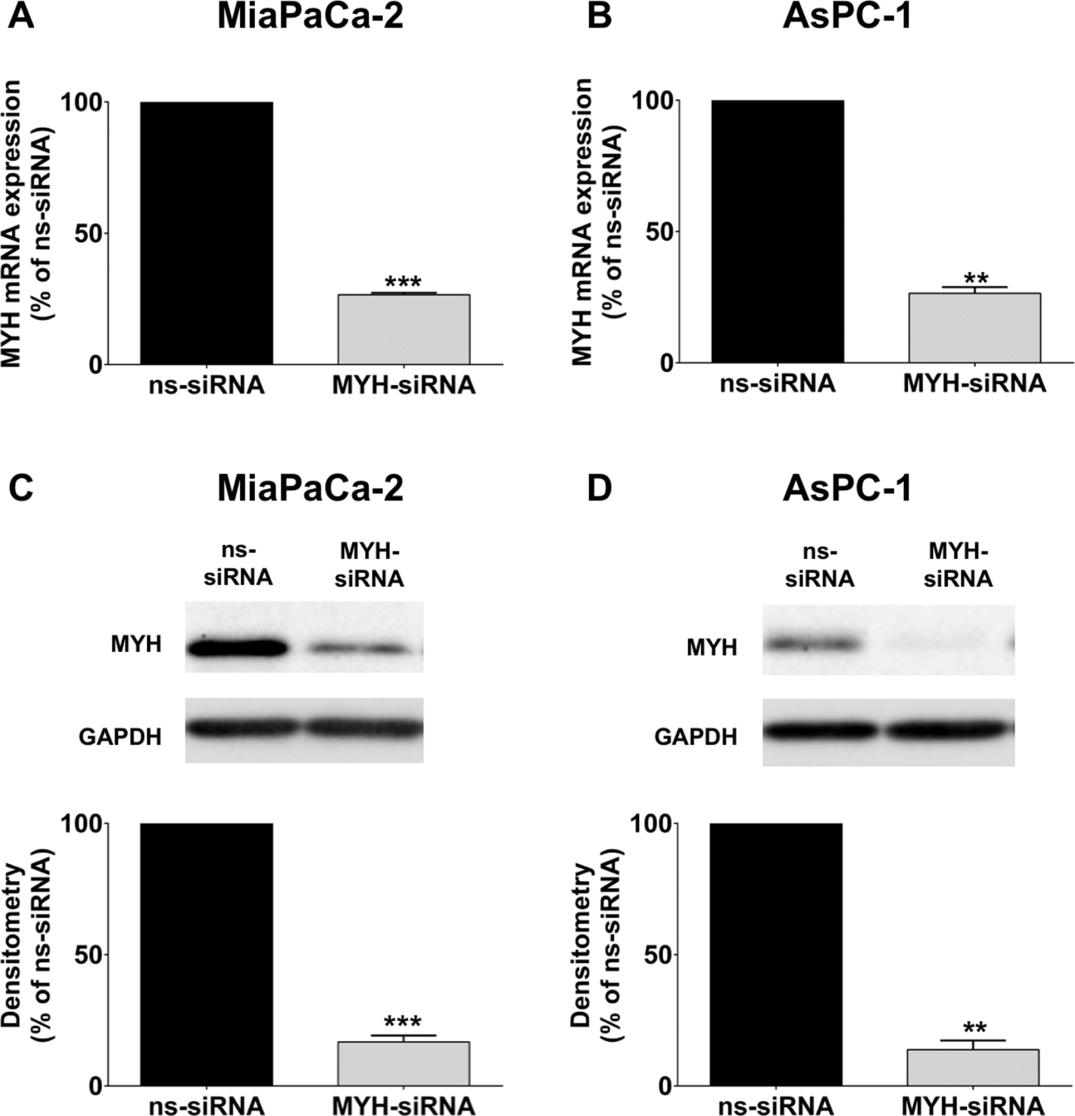
Knockdown of MYH in pancreatic cancer cells RNA and protein was extracted from cells 96 h after transfection with control siRNA (ns-siRNA) or MYH-siRNA. (**A–B**) qPCR analysis of MYH knockdown in RNA extracts from (A) MiaPaCa-2 and (B) AsPC-1. Samples were standardised to 18S RNA. (**C**–**D**) Western blot analysis of MYH silencing in protein extracts from (C) MiaPaCa-2 and (D) AsPC-1 cells. GAPDH was used as a loading control. Graphs show densitometry of Western blots for MYH (representative Western blots shown in top panel). Asterisks indicate significance (**p ≤ 0.01, ***p ≤ 0.001; *n* = 3).

We next established whether MYH could be silenced in PC cells using siRNA. MiaPaCa-2 and AsPC-1 cells were transfected with either control non-silencing siRNA (ns-siRNA) or siRNA against MYH (MYH-siRNA). At 96 h post-transfection, we observed > 80% knockdown at both the mRNA and protein level in both cell lines (MiaPaCa-2 = 83.3 ± 2.5% protein knockdown and 73.4 ± 0.8% RNA knockdown relative to ns-siRNA, *p* < 0.001; AsPC-1, 86.2 ± 3.5% protein knock-down and 73.6 ± 2.4% RNA knock-down relative to ns-siRNA, *p* < 0.01; Figure 2). To determine if knockdown of MYH resulted in a compensatory increase in OGG1, we also measured OGG1 protein expression in PC cells following treatment with MYH-siRNA. MYH knockdown had no effect on OGG1 protein expression in MiaPaCa-2 and AsPC-1 (Supplementary Figure S1).

### MYH knockdown reduces PC cell proliferation and sensitizes them to oxidative stress

**Figure 3:**
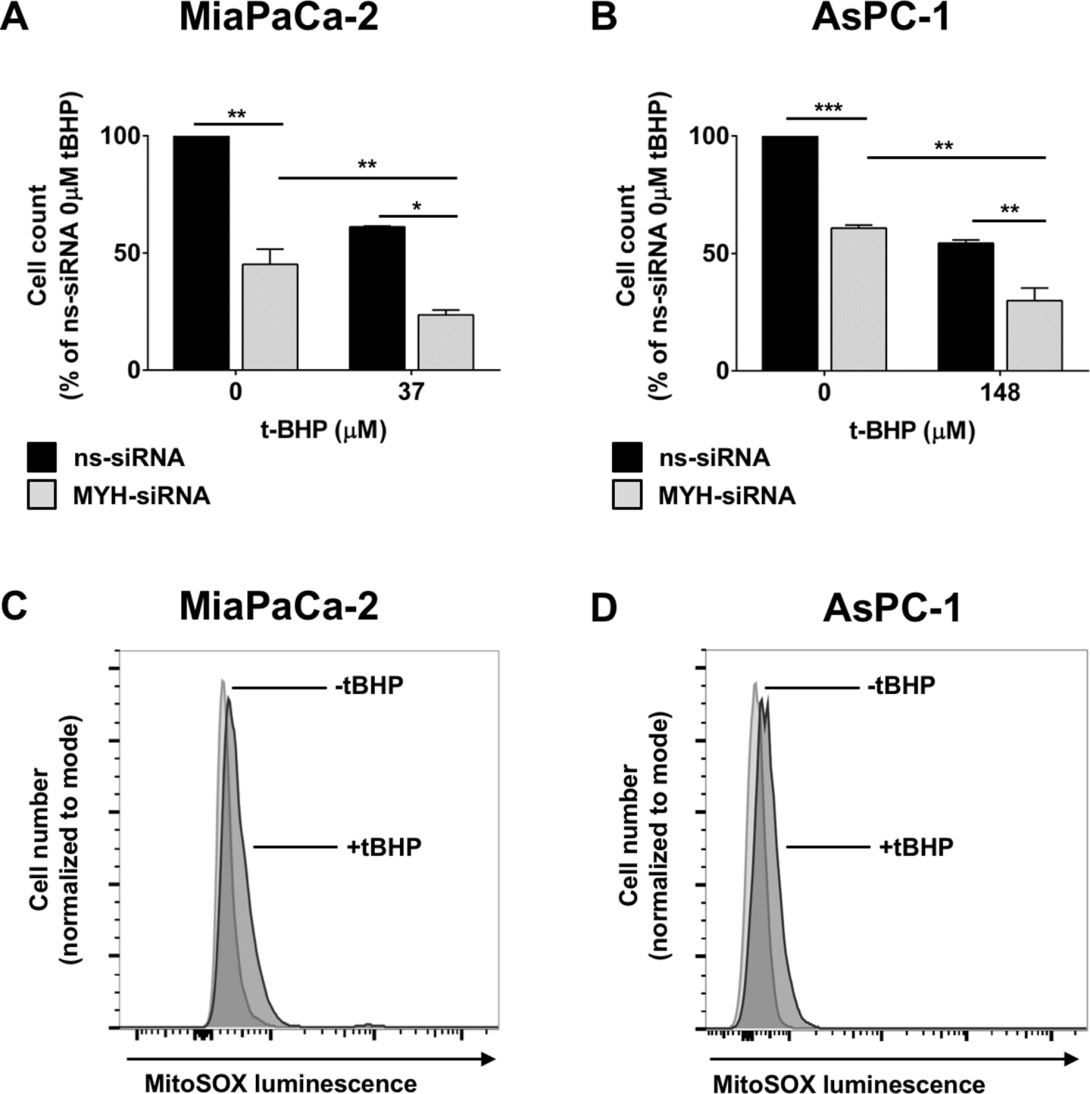
The effect of MYH knockdown on pancreatic cancer cell proliferation and sensitivity to oxidative stress (**A**–**B**) Proliferation assay of (A) MiaPaCa-2 cells and (B) AsPC-1 cells transfected with control siRNA (ns-siRNA) or MYH-siRNA. 48 h post-transfection, cells were cultured ± t-butyl hydroperxoide (t-BHP) for a further 48 h. Cells were then lifted and live cells counted on an automated BioRAD cell counter (trypan blue staining). Bars represent the total live cell count as a fraction of ns-siRNA control (± s.e.m.). (**C**–**D**) MitoSOX^TM^ assay of oxidative stress in (C) MiaPaCa-2 and (D) AsPC-1 cells after stimulation with 37 μM or 148 μM t-BHP, respectively. Asterisks indicate significance relative to ns-siRNA controls (*p ≤ 0.05, **p ≤ 0.01, ***p ≤ 0.001; *n* ≥ 3).

We then assessed the effect of MYH knockdown on PC cell proliferation under normal culture conditions. MiaPaCa-2 and AsPC-1 cells were transfected with ns-siRNA or MYH-siRNA. Proliferation was measured 96 h post-transfection, by trypan blue staining and live cell count on a BioRAD automated cell counter. MYH knockdown significantly reduced the proliferation of both PC lines 96h post-transfection, relative to ns-siRNA controls (MiaPaCa-2 = 54.8 ± 6.5% reduction relative to ns-siRNA, *p* < 0.05; AsPC-1 = 39.21 ± 1.3% reduction relative to ns-siRNA, *p* < 0.05; Figure 3A–3B). Notably, this effect was maintained when the experiment was repeated in the presence of hypoxia (48 h), a prominent feature of the PC microenvironment (Supplementary Figure S2A–S2B).

To determine if MYH was important in protecting PC cells from oxidative stress, we measured the effect of MYH knockdown on PC cell proliferation in the presence of the oxidative stress agent t-butyl hydroperoxide (t-BHP). MiaPaCa-2 and AsPC-1 cells were transfected with ns-siRNA or MYH-siRNA. 48 h post-transfection, either normal medium (control) or t-BHP was added to the cultures. Addition of t-BHP to culture medium significantly increased intracellular oxidative stress in PC cells, as assessed by MitoSOX^TM^ staining (fluoresces in response to oxidation by superoxide in mitochondria; increased intracellular oxidative stress results in increased fluorescence) and flow cytometry (Figure 3C–3D). Cells were cultured for a further 48 h before proliferation was measured by trypan blue staining and live cell count. We found that MYH knockdown was able to significantly increase the sensitivity of both PC lines to oxidative stress (Figure 3A–3B).

### Silencing MYH in PC cells modulates cell cycle and induces apoptosis

**Figure 4:**
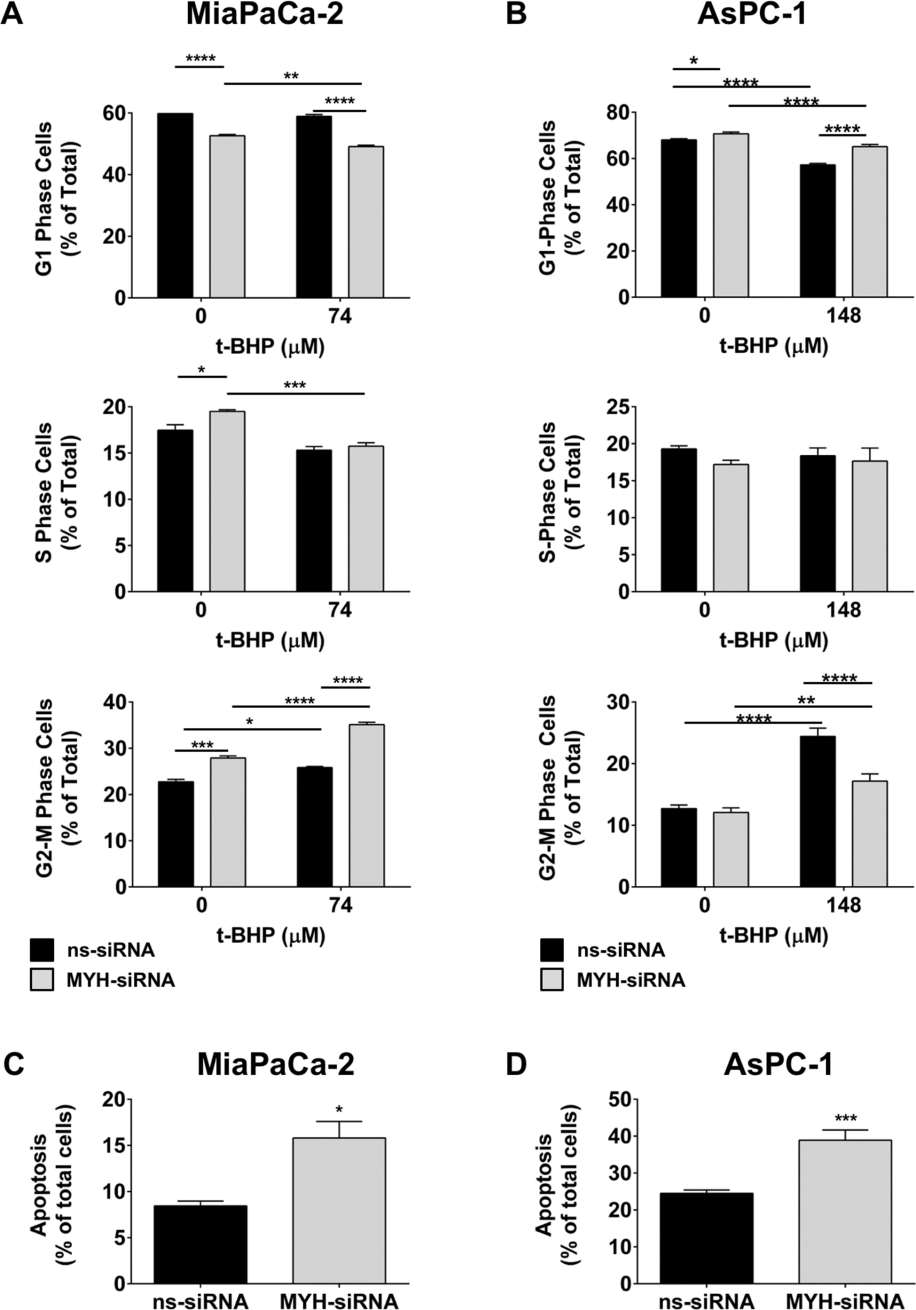
MYH knockdown alters cell cycle and induces apoptosis in pancreatic cancer cells (**A**–**B**) Cell cycle: (A) MiaPaCa-2 cells or (B) AsPC-1 cells were transfected with ns-siRNA or MYH-siRNA. 48 h post-transfection, cells were cultured in t-butyl hydroperoxide (t-BHP). 48 h later, cells were stained with PI and DNA content measured by flow cytometry. (**C**–**D**) Apoptosis: (C) MiaPaCa-2 or (D) AsPC-1 cells were transfected with ns-siRNA or MYH-siRNA. Apoptosis was determined 96 h post-transfection, by AnnexinV + 7AAD staining and flow cytometry. Bars represent the fraction of total MiaPaCa-2 or AsPC-1 cells that were apoptotic (± s.e.m.). Asterisks indicate significance relative to ns-siRNA controls (*p < 0.05, **p < 0.01, ***p < 0.001, ****p < 0.0001; *n* ≥ 3).

We next investigated the effect of MYH knockdown on (i) cell cycle and (ii) apoptosis in PC cells. MiaPaCa-2 and AsPC-1 cells were transfected with ns-siRNA or MYH-siRNA. (i) 48 h post-transfection, MiaPaCa-2 cells were treated with t-BHP for a further 48 h. Cells were then stained with propidium iodide and cell cycle distribution determined by flow cytometry. In MiaPaCa-2, MYH knockdown alone significantly reduced the proportion of cells in G1 phase (pre-replication) and increased the proportion of cells in S (replication) and G2/M phase (post-replication/mitosis phase; Figure 4A; Supplementary Figure S3A). Moreover, t-BHP treatment further increased the proportion of cells in G2/M phase, while reducing cells in G1- and S-phase (Figure 4A; Supplementary Figure S3A). In contrast, silencing MYH in AsPC-1 increased cells in G1-phase (Figure 4B; Supplementary Figure S3B). Treatment of AsPC-1 with t-BHP caused G2/M arrest as observed in MiaPaCa-2 (Figure 4B; Supplementary Figure S3B). However, MYH knockdown in AsPC-1 inhibited t-BHP-induced G2/M arrest (Figure 4B; Supplementary Figure S3B); (ii) 96 h post-transfection, apoptosis was measured in MiaPaCa-2 and AsPC-1 cells by AnnexinV/7AAD staining and flow cytometry. MYH knockdown significantly increased apoptosis in both PC cell lines (MiaPaCa-2: 87.4 ± 21% increase relative to ns-siRNA controls, *p* < 0.05; AsPC-1: 58.7 ± 11.2% increase relative to ns-siRNA controls, *p* < 0.001; Figure 4C–4D). The results indicating that, at least in part, the anti-proliferative effect of MYH-siRNA was via induction of apoptosis, albeit by potentially different pathways as suggested by cell cycle data.

### Silencing MYH in PC cells reduces clonogenicity and increases chemosensitivity

**Figure 5:**
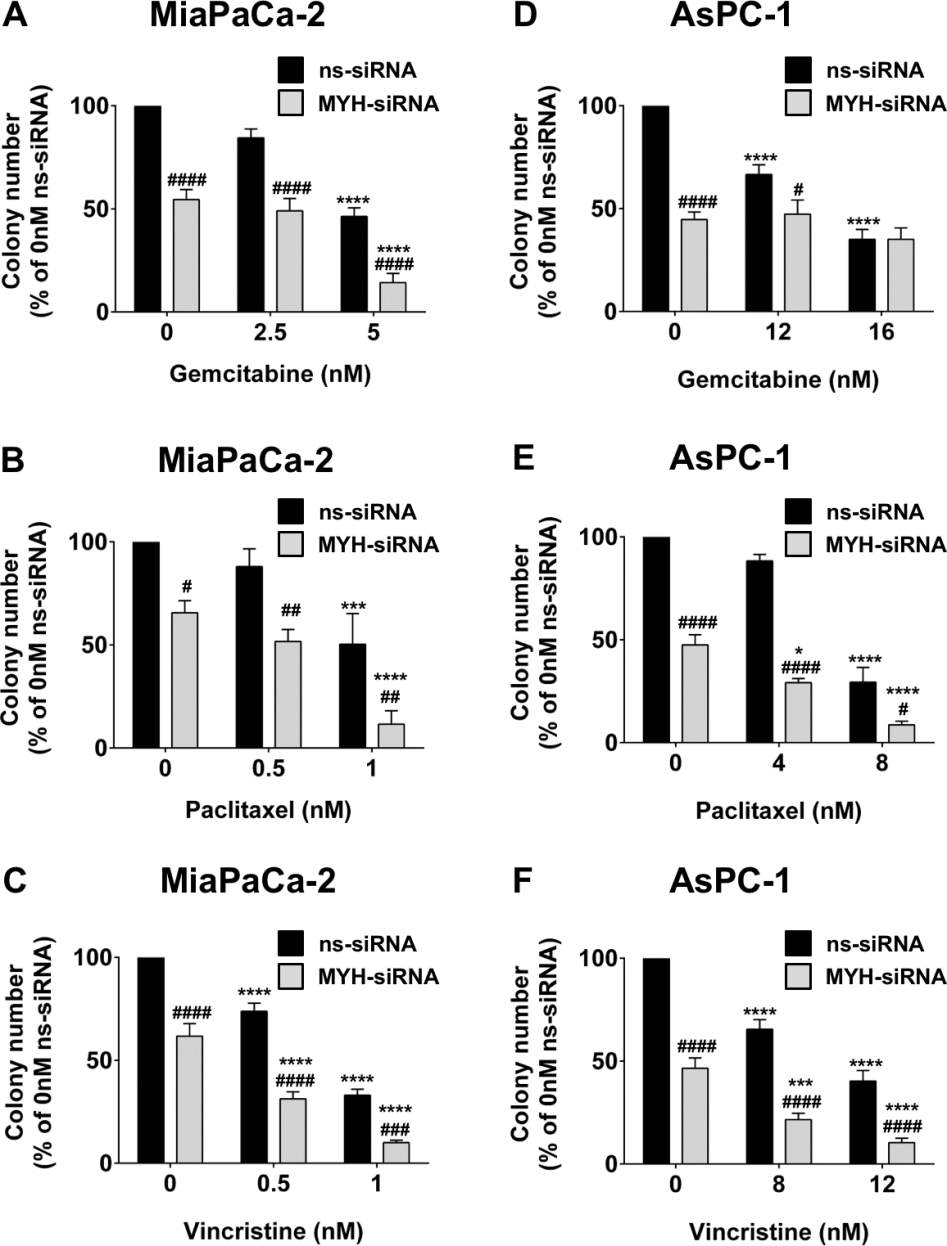
MYH knockdown reduces pancreatic cancer cell clonogenicity and increases chemosensitivity Bars represent the number of MiaPaCa-2 colonies (mean+s.e.m. as a % of control siRNA (ns-siRNA) 0 nM drug) that formed from low density seeding following transfection with ns-siRNA or MYH-siRNA and 72 h culture in gemcitabine (**A**), paclitaxel (**B**) or vincristine (**C**). (**D**–**F**) as per A–C, except experiments were carried out with AsPC-1 cells. Asterisks indicate significance relative to the 0nM control for the same siRNA (*p ≤ 0.05, ***p ≤ 0.001, ****p ≤ 0.0001; *n* ≥ 4). Hashes indicate significance relative to the ns-siRNA control of the same drug dose (^#^*p* ≤ 0.05, ^##^*p* ≤ 0.01, ^###^*p* ≤ 0.001, ^####^*p* ≤ 0.0001; *n* ≥ 4).

Chemotherapeutics are capable of causing oxidative stress in addition to their primary function [13]. We next measured the effect of silencing MYH in PC cells on chemosensitivity to the anti-metabolite gemcitabine and the microtubule binding agents paclitaxel and vincristine, using a clonogenic assay. First, we observed that MYH knockdown *alone* significantly reduced colony numbers in MiaPaCa-2 and AsPC-1 (Figure 5). Importantly, MYH knockdown potently increased the chemosensitivity of both cell lines to paclitaxel and vincristine (Figure 5B–5F) and of MiaPaCa-2 to gemcitabine (Figure 5A), relative to controls. MYH knockdown had no effect on gemcitabine sensitivity in AsPC-1 cells (Figure 5D).

### Silencing MYH in PC cells reduces their metastatic potential

**Figure 6:**
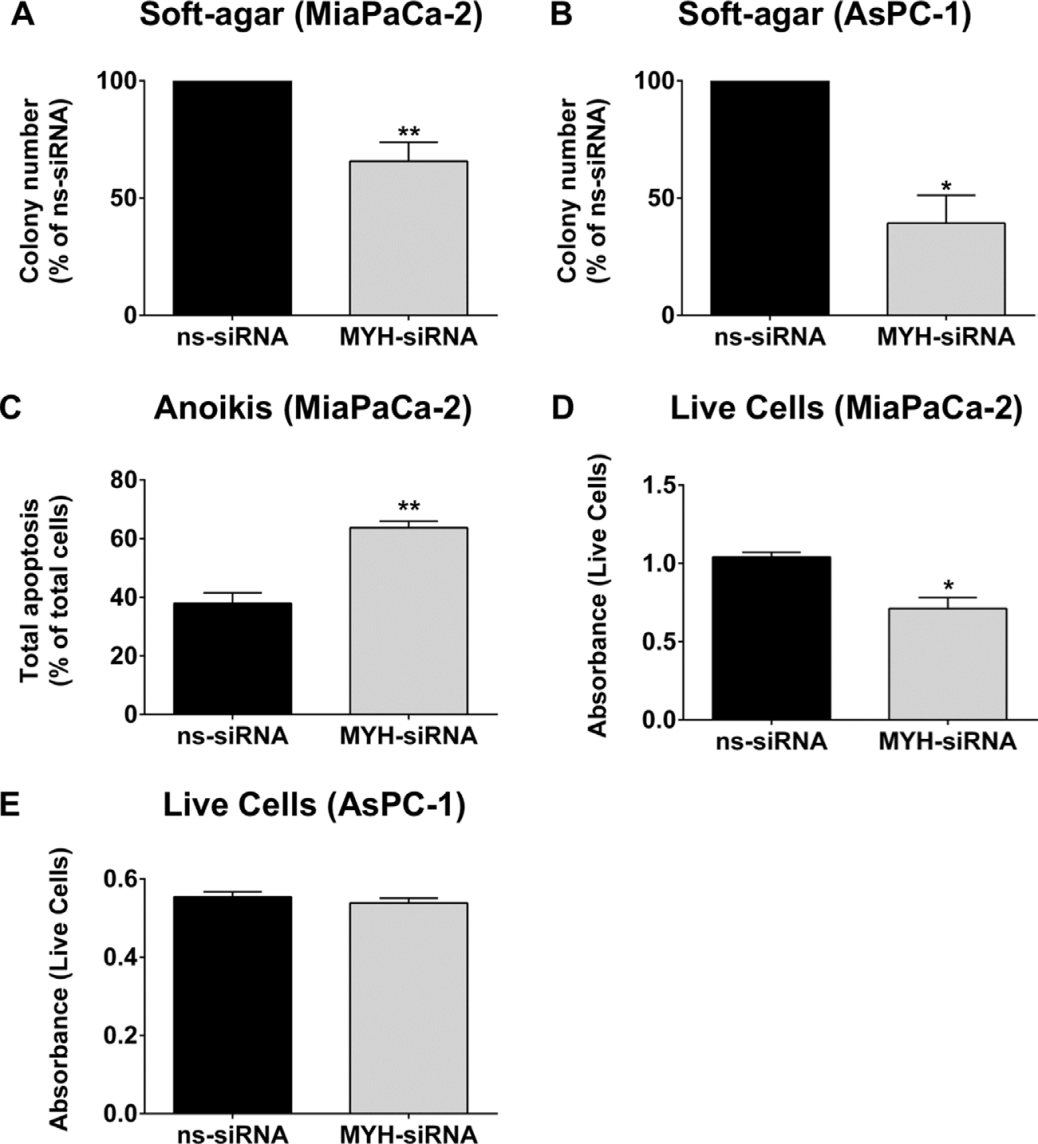
MYH knockdown reduces pancreatic cancer cell metastatic potential (**A**) MiaPaCa-2 or (**B**) AsPC-1 transfected with control siRNA (ns-siRNA) or MYH-siRNA were embedded in soft-agarose at 48 h post-transfection and allowed to form colonies. Bars represent the number of colonies that formed (mean+s.e.m. as a % of ns-siRNA). Asterisks indicate significance (*p ≤ 0.05, **p ≤ 0.01; *n* ≥ 3). (**C**) MiaPaCa-2 cells were transfected with ns-siRNA or MYH-siRNA and 24 h post-transfection cells were cultured under anchorage independent conditions for a further 48 h (wells coated with Poly-HEMA). Bars represent the apoptotic fraction determined by AnnexinV + 7AAD staining and flow cytometry (mean + s.e.m.). Asterisks indicate significance (**p ≤ 0.01; *n* = 3). (**D**–**E**) MiaPaCa-2 or AsPC-1 cells were cultured as in C), before the number of live cells was measured using cell counting kit 8 (cck8; colorimetric assay whose absorbance directly correlates with live cell number). Bars represent the absorbance for each siRNA treatment (mean + s.e.m). Asterisks indicate significance (*p ≤ 0.05; *n* = 3).

To determine if MYH also controlled the metastatic potential of PC cells, we measured the effect of MYH knockdown on anchorage-independent survival using (i) a soft-agar assay and (ii) an anoikis (anchorage-independent apoptosis) assay. (i) PC cells were transfected with ns-siRNA or MYH-siRNA and seeded into soft-agar. 3D colonies (> 50 cells) were allowed to form, and were then stained with MTT reagent and counted. MYH knockdown significantly reduced colony number of both PC cell lines, relative to ns-siRNA controls (MiaPaCa-2: 34.3 ± 8% reduction in colony number; AsPC-1: 65.4 ± 14.1% reduction in colony number; *p* < 0.05; Figure 6A–6B). (ii) MiaPaCa-2 cells were transfected with ns-siRNA or MYH-siRNA, then cultured for 48 h in polyHEMA-coated plates (in suspension). Apoptosis was then measured by AnnexinV/7AAD staining and flow cytometry. We found that MYH knockdown significantly increased anoikis in MiaPaCa-2 cells, relative to ns-siRNA controls (67.9 ± 5.8% increase relative to ns-siRNA controls, *p* < 0.01; Figure 6C). We then performed a complimentary assay suitable for measuring anchorage-independent survival in MiaPaCa-2 and AsPC-1 cells under the same conditions, using cell counting kit 8 (Dojindo). This colorimetric assay is a measure of the number of live cells in a culture. Consistent with anoikis assay results, MYH knockdown in MiaPaCa-2 reduces the number of live cells under anchorage independent growth (Figure 6D). However, no effect was observed in AsPC-1 cells (Figure 6E), suggesting the effect of MYH knockdown on PC cell anoikis is cell line –specific.

### MYH knockdown reduces subcutaneous PC tumor growth

**Figure 7:**
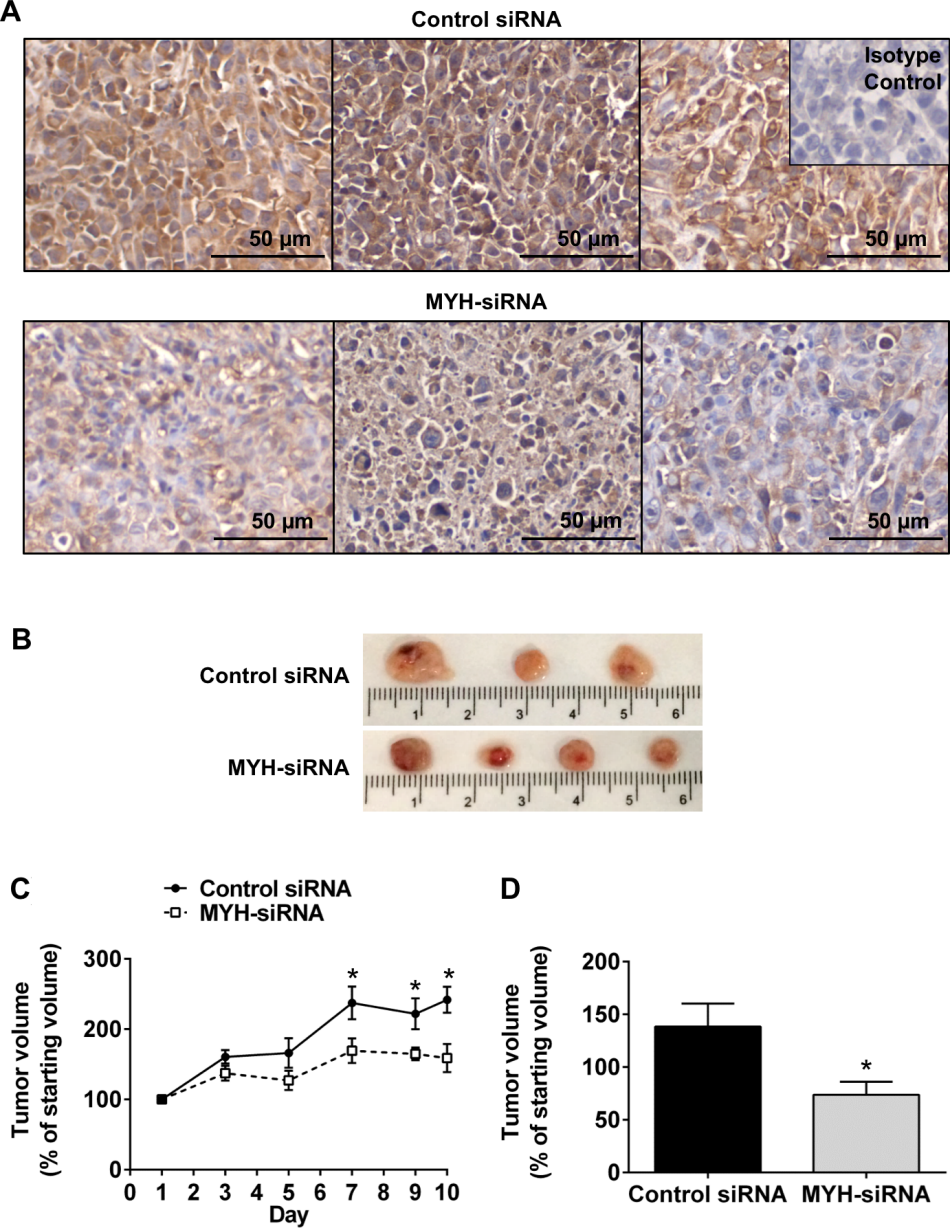
Silencing MYH in pancreatic cancer cells reduces subcutaneous tumor growth (**A**) Immunohistochemistry for MYH in subcutaneous pancreatic (MiaPaCa-2) tumor sections treated with control siRNA or MYH-siRNA. (**B**) Representative photomicrographs of subcutaneous tumors treated with control siRNA or MYH-siRNA. (**C**) Growth curves showing tumor volume, over the course of JetPEI^®^ + control siRNA or MYH-siRNA treatments (mean ± s.e.m.). (**D**) Tumor volumes, as a fraction of starting volume, calculated from *ex vivo* measurements at endpoint (excised tumors). Asterisks indicate significance (*p ≤ 0.05; *n* = 4 for control siRNA and *n* = 6 for MYH-siRNA).

We tested whether the effects of MYH silencing in PC cells would translate to an *in vivo* setting, using a subcutaneous mouse model of PC (MiaPaCa-2). Control- or MYH-siRNA was delivered intratumorally every 48 h using *in vivo* JetPEI^®^ (Polyplus), a nanoparticle designed for *in vivo* siRNA delivery and currently in clinical trial for the treatment of PC (clinicaltrials.gov Identifier: NCT01274455). Tumor volume was calculated every 48 h by calliper measurement. Tumors were harvested 24 h after the final injection for immunohistochemical analysis of MYH knockdown. We confirmed MYH knockdown in PC tumors (Figure 7A) and observed that MYH knockdown significantly reduced tumor growth, relative to controls (Figure 7B–7D).

## DISCUSSION

PC is a highly lethal disease that urgently requires more effective therapeutic treatments. There is strong rationale for targeting proteins that protect cells from oxidative stress in solid tumors like PC, as the hypoxic tumor microenvironment, the altered metabolism of tumor cells and chemotherapeutic treatments can all increase intracellular oxidative stress [6, 7, 9, 12, 13]. MYH is a key DNA repair protein involved in repairing DNA damage caused by oxidative stress [22]. Few studies have investigated the functional role of MYH in cancer cells and are conflicting on whether inhibition of MYH protects cancer cells or compromises their survival in oxidative stress. For example, Hwang et al. [28] demonstrated that MYH knockdown in HeLa cells sensitizes them to oxidative stress, whereas a more recent study in multiple cancer cell lines by Oka et al. [29] showed that MYH knockdown improved cancer cell survival in p53-proficient cells. In this manuscript, for the first time, we identified MYH as an important survival factor in PC cells (p53-mutated). Using RNAi, we showed that MYH knockdown in PC cells significantly reduced their tumorigenic and metastatic potential and increased their sensitivity to a broad range of chemotherapeutic drugs.

We started our investigation by examining MYH protein levels in human PC tissue specimens. Current International Cancer Genome Consortium databases show that mutations of MYH and alterations in mRNA levels occur in < 4% of patients with PC (based on Californian and Australian PC databases–International Cancer Genome Consortium). However, MYH protein levels have never been investigated in PC. We found that MYH was upregulated in human PC tissue, relative to normal/benign pancreas conditions. When we examined MYH protein levels in HPDE cells and different PC cell lines, we observed significant variation in expression, reflecting the heterogeneity of PC. The high level of MYH in HPDE cells was somewhat unexpected given the low level of staining in human control pancreas tissue. However, rapidly proliferating HPDE cells cultured on plastic do not necessarily reflect the status of these cells *in vivo*. Moreover, an examination of MYH mRNA expression in various tissues in the body showed low MYH expression in normal pancreas and highest expression in the thymus [30].

To represent the clinical situation, we pursued our experiments in PC cells of different origins (MiaPaCa-2: primary tumor-derived; AsPC-1: metastatic site–derived) and with different levels of MYH expression (MiaPaCa-2: higher MYH expression; AsPC-1: lower MYH expression). Despite the differences in MYH expression between the cell lines, MYH silencing significantly decreased the proliferation of both MiaPaCa-2 and AsPC-1 cells in the absence of any additional stress. This effect was enhanced by the addition of t-BHP, an oxidative stress-inducing compound. The results suggested that MYH plays a basic survival function in PC cells, that is likely associated with its established function in maintaining genomic integrity in the presence of oxidative stress [28]. In contrast, hypoxia failed to enhance the anti-proliferative effect of MYH knockdown. However, it should be noted that our *in vitro* system is a model of acute hypoxia, which may not have the same effect as chronic hypoxia and does not generate additional oxidative stress (Supplementary Figure S2C). What this result did highlight was that the effect of MYH knockdown was maintained in the presence of a key feature of the PC microenvironment.

Further evidence supporting MYH's proposed function in PC cells came from our cell cycle analysis. In MiaPaCa-2 cells, MYH knockdown alone significantly increased accumulation of cells in replication and post-replication phases (when DNA is most exposed to free radicals) while reducing the proportion of cells in G1 phase. The altered cell cycle may be indicative of increased DNA damage that now has to be repaired by less efficient DNA repair pathways, or that is triggering checkpoint arrest. Moreover, t-BHP further increased accumulation of cells in G2-M phase, suggesting hindered G2-M phase progression or arrest. In contrast, MYH knockdown in AsPC-1 cells increased accumulation of cells in G1-phase. Addition of t-BHP hindered G2-M phase progression as in MiaPaCa-2 cells. However, unlike MiaPaCa-2, MYH knockdown in AsPC-1 did not enhance G2-M phase arrest, but in fact reduced it. The results implied that MYH knockdown may have induced different mechanisms in each line. Based on MYH's well-established DNA repair role, it is likely that MYH knockdown allowed greater accumulation of oxidant-induced mutation in both cell lines. In MiaPaCa-2, this appeared to have triggered a checkpoint response, whereas in AsPC-1, there may have been a failure to trigger a checkpoint response. Regardless of the mechanism, MYH knockdown in significantly increased apoptosis in both of the tested PC lines. The results suggest that MYH may be a versatile target in PC cells. On the one hand, it can hinder cell cycle progression, potentially leading to or accompanying apoptosis, while on the other, it can suppress cell cycle responses to DNA damage, leading to unchecked accumulation of DNA damage and ultimately apoptosis. Indeed, MYH has been shown to control cell cycle checkpoint proteins in response to DNA damage through intermediate signalling proteins [31, 32]. Differences in these proteins between different PC cell lines may dictate the pathway by which MYH knockdown ultimate leads to cell death.

Chemotherapeutic agents are also known to induce oxidative stress [13]. We investigated whether MYH silencing could increase the sensitivity of PC cells to the first-line PC treatments gemcitabine (anti-metabolite) and paclitaxel (tubulin stabilizer) and to an additional tubulin binding agent, vincristine (tubulin destabilizer), using a clonogenic assay. Gemcitabine and paclitaxel were chosen for their direct clinical relevance, as first-line treatments for pancreatic cancer. Vincristine was included as an additional structurally and functionally distinct chemotherapeutic. This was to help discern if the effects observed with paclitaxel were drug-specific. MYH knockdown alone was able to significantly reduce colony formation in both PC lines, re-iterating MYH's basic survival function in these cells. MYH knockdown also increased the chemosensitivity of the tested lines to tubulin binding agents (paclitaxel and vincristine) and of MiaPaCa-2 to the anti-metabolite gemcitabine. The results suggest that MYH inhibition could increase the efficacy of a broad range of chemotherapeutics. However, the cell-specific sensitization of MiaPaCa-2 cells to gemcitabine also suggests that MYH may be playing roles beyond its glycosylase activity, which may vary between cells depending on additional proteins it interacts with. In addition, tubulin binding agents and nucleotide/nucleoside analogs generate relatively low levels of oxidative stress [13], supporting the proposition that the observed chemosensitization effects may also be due to other functions of MYH.

PC is a highly metastatic disease, and any effective therapies against this cancer need to target the metastatic process. MYH has never been shown to be involved in any aspect of the metastatic process. However, we found that MYH knockdown significantly reduced anchorage-independent growth of PC cells. In MiaPaCa-2 cells, this effect was a result of increased induction of anoikis (anchorage-independent apoptosis), which is resisted by metastatic cancer cells [33]. However, in AsPC-1 we did not observe increased anoikis, suggesting that these effects are dependent on the cell line. This does not detract from the fact that MYH knockdown reduced the anchorage-independent growth of both PC lines. Our promising results indicate that, in addition to anti-tumorigenic effects and chemosensitization, MYH inhibition might inhibit the establishment of metastatic PC nodules in an *in vivo* setting.

While our *in vitro* assays provided strong evidence that MYH may be effective in a therapeutic setting, they lacked the complexity of an *in vivo* setting. We tested the effect of MYH-siRNA on PC tumor growth in an *in vivo* setting, using a subcutaneous PC mouse model and the nanoparticle JetPEI^®^ for siRNA delivery. JetPEI^®^ complexed to plasmid DNA encoding a fusion protein that converts gemcitabine to its toxic metabolite, is currently in clinical trial for PC (clinicaltrials.gov Identifier: NCT01274455). Consistent with our *in vitro* findings, silencing MYH in PC cells suppressed tumor growth. These results strengthen our proposal that MYH may be a therapeutic target for PC and form the foundation for future testing in orthotopic PC mouse models that combine PCs and pancreatic stellate cells (key pro-fibrogenic/pro-tumorigenic cells) in an *in vivo* setting.

Any approach that systemically targets a DNA repair protein does carry some risks: (1) the potential for toxicity in normal cells of the body; (2) the potential to generate cancer elsewhere in the body; (3) generation of drug-resistant cancer cells. An approach targeting MYH would ideally be combined with chemotherapeutics that synergise with MYH, enhancing their ability to kill PC cells and reducing the chances of leaving resistant cancer cells. Off-target toxicity and mutation could also be minimised using a cancer cell-specific delivery method. This is important in the case of MYH as mutations in this protein are known to cause colon cancer [34]. Moreover, MYH knockout mice, while viable, are 1.7 times more likely to develop spontaneous tumours, particularly in the intestines [35]. On this point, siRNA therapeutics coupled to cancer-targeting nanoparticles represent an ideal avenue for targeting MYH in PC cells, while overcoming the current lack of pharmacological inhibitors against MYH [36–38].

Our novel work has identified MYH as a potential therapeutic target for PC that can (i) decrease PC cell survival, (ii) improve the efficacy of existing therapeutics, including first-line treatments for PC (gemcitabine/paclitaxel) and (iii) reduce the metastatic potential of PC cells.

## MATERIALS AND METHODS

### Cell culture

Human PC cells (MiaPaCa-2, Panc-1, HPAFII and AsPC-1) were obtained from the American Tissue Culture Collection. MiaPaCa-2 cells were maintained in DMEM culture media, supplemented with 10% fetal bovine serum (FBS), 2.5% horse serum and 2 mM L-glutamine. Panc-1 cells were cultured in DMEM culture media, supplemented with 10% fetal bovine serum (FBS) and 2 mM L-glutamine. HPAFII cells were cultured in MEM culture media supplemented 10% fetal bovine serum (FBS), 2 mM L-glutamine and 1mM sodium pyruvate. AsPC-1 cells were cultured in RPMI culture media supplemented with 10% FBS and 2 mM L-glutamine. All cells were grown at 37°C in a humidified incubator with 5% CO_2_, and were routinely screened and found to be free of mycoplasma. Purity of cell lines was confirmed by short tandem-repeat profiling (CellBank Australia). Normal Human Pancreatic Ductal Epithelial (HPDE) cells (a kind gift from Ming Tsao, Ontario Cancer Institute) were grown in Keratinocyte serum-free (KSF) medium supplemented with 50 mg/ml bovine pituitary extract (BPE) and 5 ng/ml epidermal growth factor (EGF).

### Immunohistochemistry of human PC tissue specimens

Human PC tissue specimens were collected by surgical removal. The use of human PC tissue sections was approved by the UNSW Human Research Ethics Committee (HCEC# HC14039). Paraffin-embedded tissue sections were stained with MYH antibody (1:100; Abnova) as previously described [39]. 3,3′ diaminobenzidine (DAB) was used as the substrate and sections were counterstained using hematoxylin. The specificity of the primary antibodies was confirmed by including isotype control antibodies at the same concentration as the primary antibodies.

### siRNA transfection

PC cells were transfected with siRNAs at a final concentration of 100 nM using Lipofectamine 2000 (Invitrogen) according to the manufacturer's instructions. All cells were transfected with smart pool On-Target Plus (Thermoscientific) siRNAs, which contained a pool of four different individual siRNAs that target different regions of the specific gene. siRNAs designed against MYH (Thermoscientific, Cat. L-008260-00L-020099-00) or non-targeting (control; Cat. D-001810-10-20) were used for this study. Knockdown was assessed 96h post-transfection by qPCR and Western blot.

### Quantitative real-time PCR

Total RNA was extracted from pancreatic cancer cells and transcribed to cDNA using a High-Capacity cDNA Reverse Transcription Kit (Applied Biosystems). qPCR was performed using the QuantiFast SYBR Green PCR kit (Qiagen) as previously described [39]. MYH primers were obtained from Qiagen (Cat. QT00039739; Quantitect Primer Assay, Qiagen). All data were normalized to the 18S gene (Cat. QT00199367; Quantitect Primer Assay, Qiagen).

### Western blot analysis

Whole cell lysates were prepared and western blot analysis was performed using the following antibodies: HIF1-alpha (Becton Dickinson Bioscience), MYH (Abnova), OGG1 (Abcam Ltd) and GAPDH (Abcam Ltd) as previously described [40]. The blots were scanned using LAS4000 scanner and quantified using ImageQuant TL (GE Healthcare).

### Cell proliferation assays

For hypoxia experiments, cells were incubated in a hypoxic chamber (1% O_2_, 5% CO_2_, 94% N_2_) for the final 48 h of the assay. Control cells were cultured under normal conditions (atmospheric O_2_). Induction of hypoxia was confirmed by Western blot for HIF1-alpha using whole cell protein extracts. For oxidative stress experiments, cells were cultured in the presence or absence (control) of 37–148 μM t-butyl hydroperoxide (t-BHP) for the final 48 h of the assay. t-BHP concentrations were selected based on an ability to reduce PC cell proliferation by 50% after 48 h and to induce intracellular oxidative stress (determined by MitoSOX^TM^ assay). Cells were trypsinised 96 h post-transfection and resuspended 1:1 in culture medium and trypan blue staining solution. Proliferation was measured by counting live cells (trypan blue negative) using a BioRAD automated cell counter. Alternatively, cells were stained for BrdU using a BrdU-APC kit (Becton Dickinson) according to the manufacturer's instructions. BrdU incorporation was analysed by flow cytometry.

### Detection of oxidative stress

Oxidative stress was confirmed by MitoSOX^TM^ Red reagent (Life technologies) staining and flow cytometry, according to the manufacturer's instructions. Briefly, cells were incubated in t-butyl hydroperoxide for 48 h, with 24 h top-up, before incubation with MitoSOX^TM^ Red reagent for 10 minutes. MitoSOX^TM^ fluoresces in response to oxidation by superoxide in mitochondria. The amount of fluorescence produced is thus a measure of intracellular oxidative stress.

### Cytotoxic drug preparation

Gemcitabine (Hospira) was prepared at a stock concentration of 126.8 mM in saline. Paclitaxel (Calbiochem, Merck Biosciences) was prepared at a stock concentration of 2 mM in DMSO. Vincristine (Sigma) was prepared at a stock concentration of 2 mM in DMSO.

### Cytotoxic drug-clonogenic assays

24 h post-siRNA transfection cytotoxic drug-clonogenic assays were performed as previously described [40]. Briefly, cells were seeded at low density into 6-well plates (MiaPaCa-2 = 300 cells/well; AsPC-1 = 500 cells/well). 24 h post-seeding, cells were incubated in gemcitabine, paclitaxel or vincristine for a total of 72 h. Colonies were allowed to form 7–12 days post-incubation in cytotoxic drugs. Colonies (≥ 50 cells) were then stained with crystal violet and counted.

### Cell cycle analysis

48 h post-transfection with siRNA, cells were incubated with or without 74 μM t-BHP for a further 48 h. Cells were then lifted and fixed in 80% ice-cold ethanol for 30 min. After fixation, cells were stained with 10 μg/ml Propidium Iodide (PI) in staining buffer (PBS/1% TWEEN-20/4 μg/ml RNase-A) for 40 min at 37°C. DNA content was assessed on a BD FACSCanto-II flow cytometer.

### Detection of apoptosis

PC cells were transfected with siRNA as described above. 96 h post-transfection, both adherent and floating cells were collected and cell death was measured using the AnnexinV-PE/7-AAD reagent (Becton Dickinson AnnexinV-PE/7AAD Apoptosis Kit) according to the manufacturer's instructions. Samples were analysed on a BD FACSCanto-II flow cytometer.

### Soft-agar assay

48 h post-transfection control cells (ns-siRNA) were counted and seeded at 2000 (MiaPaCa-2) OR 5000 (AsPC-1) cells per well. All other transfections were seeded according to control cell seeding volume. PC cells were seeded in 0.33% agar in 2 × growth medium (2 × concentration of all supplements) on a 5% agar layer in 2 × growth medium, in 6 well plates. Colonies (≥ 50 cells) were allowed to grow over 2 weeks, after which plates were stained with 5mg/ml MTT in PBS for 30-60 minutes and visualized on an ImageQuant LAS4000 luminometer (GE Healthcare). Colony counting was carried out using the automated colony counting module of ImageQuant analysis software.

### Anoikis assay

A stock solution of poly 2-hyroxyethyl methacrylate (Poly-HEMA; 120 mg/ml) was prepared in 95% ethanol and further diluted 1:10 in 95% ethanol prior to use. 1 ml of this solution was pipetted into a 6-well tissue culture plate and left to dry for 24 h at room temperature. Prior to use, wells were washed with PBS and complete cell culture medium. 24 h post-siRNA transfection MiaPaCa-2 cells were seeded into the Poly-HEMA-coated wells. Cell death was then measured 48 h later by annexin V/7AAD staining as described above. Alternatively, MiaPaCa-2 cells and AsPC-1 cells were cultured under the above conditions before the number of live cells were measured using cell counting kit 8 (Dojindo).

### Subcutaneous PC mouse model

8 week old female BALB/c nude mice were used. All animal experiments were approved by the Animal Ethics committee, UNSW (ACEC 15/99B). MiaPaCa-2 cells (4 × 10^6^) were injected in 100 μl PBS into the right flank of host mice. 16 days post-implant, mice were injected intratumourally with 20 ug of control siRNA (Cat. P-002048-01, Millenium Science) or MYH-siRNA (5′CGG AAG AGG UGG UAU UGC A 3′) complexed at 8:1 N/P ratio with *in vivo* JetPEI^®^ (Polyplus), every 48 h for a total of 5 injections. Tumor volumes were calculated using the formula (length × width × height)/2. Mice were randomized based on tumor volume. Average tumor volume at the start of treatments was 53.9 ± 6.1 mm^3^. Tumors were measured using callipers every 48 h. Mice were sacrificed 24 h after the final treatment and tumors were harvested for immunohistochemistry.

### Immunohistochemistry of mouse subcutaneous PC tumors

Paraformaldehyde-fixed/paraffin-embedded tissue sections were stained with MYH antibody (1:100, overnight, 4°C; Abnova) using a mouse-on mouse kit (Abacus ALS) according to the manufacturer's instructions. 3,3′ diaminobenzidine (DAB) was used as the substrate and sections were counterstained using hematoxylin. The specificity of the primary antibodies was confirmed by including isotype control antibodies at the same concentration as the primary antibodies.

### Statistical analyses

Data are expressed as mean ± SE and were analyzed using Student *t-test* or ANOVA followed by nonparametric Dunnett. Statistical analyses were performed using GraphPad Prism. A *P value* of < 0.05 was considered statistically significant.

### Statement of significance

This study reports for the first time that silencing the DNA repair protein MutY-Homolog in pancreatic cells *in vitro:* 1) reduced pancreatic cancer cell proliferation/clonogenicity and increased apoptosis; 2) significantly sensitized pancreatic cancer cells to a broad range of chemotherapy drugs; and 3) decreased pancreatic cancer cell metastatic potential. Importantly, therapeutic administration of MYH-siRNA intratumorally using a clinically relevant nanoparticle demonstrated reduced pancreatic tumor growth *in vivo*.

## SUPPLEMENTARY MATERIALS FIGURES


