# Supplementary material for: MutY-Homolog (MYH) inhibition reduces pancreatic cancer cell growth and increases chemosensitivity
Source: Oncotarget. 2016 Dec 16;8(6):9216–29. doi: 10.18632/oncotarget.13985 (PMC5354726; doi:10.18632/oncotarget.13985)
Supplement: Supplementary file 1 [file oncotarget-08-9216-s001.pdf]

MutY-Homolog (MYH) inhibition reduces pancreatic cancer cell growth and increases chemosensitivity

Supplementary Materials

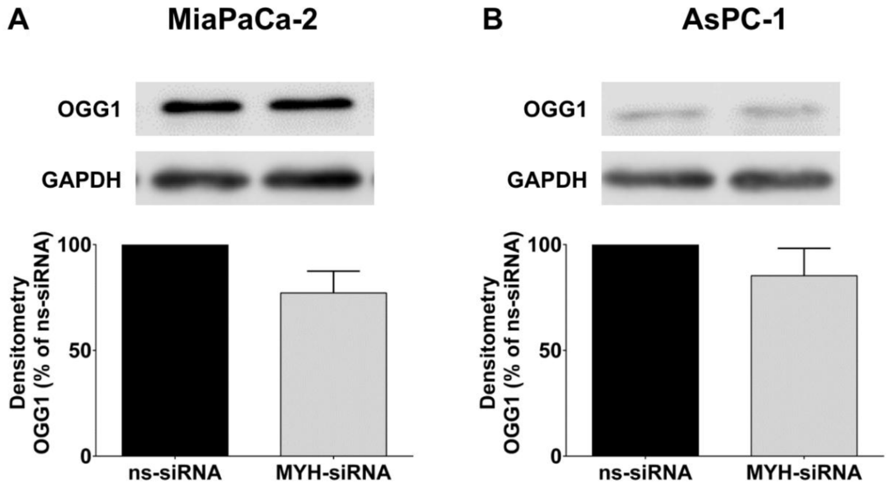

Supplementary Figure S1: MYH knockdown does not affect OGG1 expression in PC cells. Cell lysates were harvested 96 h after transfection with control siRNA (ns-siRNA) or MYH-siRNA, from (**A**) MiaPaCa-2 or (**B**) AsPC-1 cells. OGG1 was then detected by Western blot. GAPDH was used as a loading control. Graphs show densitometry analysis of OGG1 protein bands standardised to GAPDH.

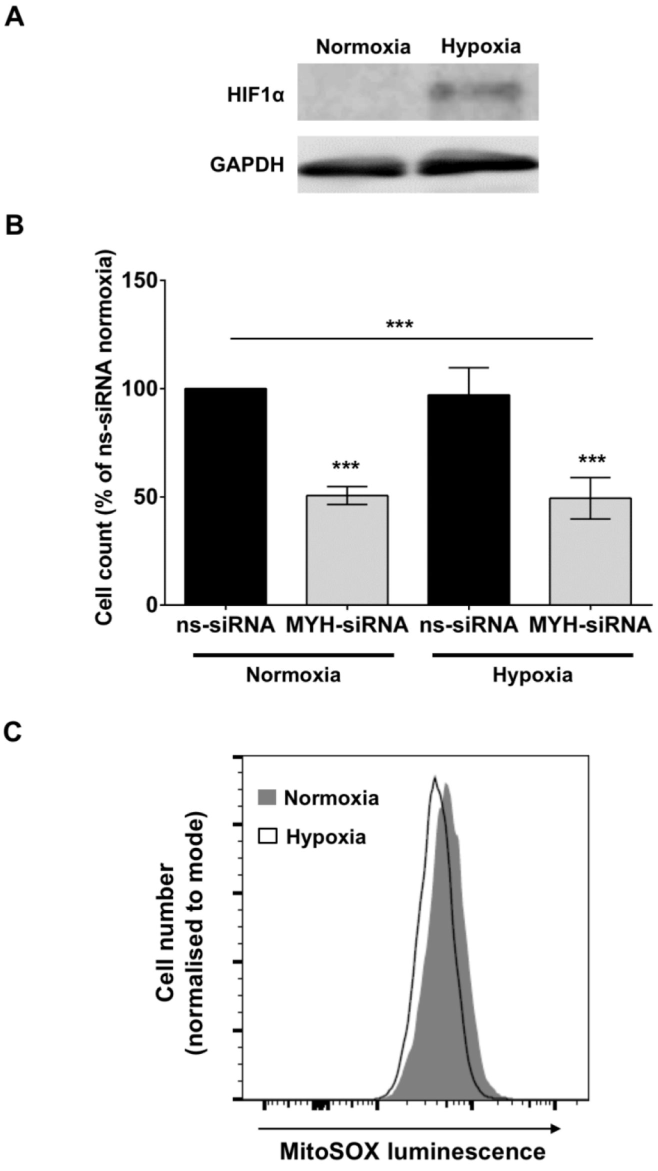

Supplementary Figure S2: The anti-proliferative effect of MYH knockdown in PC cells is maintained in the presence of hypoxia. At 48 h post-transfection, cells were cultured in a hypoxic chamber (hypoxia; 1% O_2, 5% CO_2, 94% N_2) or under normal culture conditions (normoxia; atmospheric O_2) for a further 48 h. (**A**) Western blot analysis for HIF1-alpha in cells cultured under normoxia or hypoxia. GAPDH was used as a loading control. (**B**) Proliferation assay. Cells were lifted and counted by flow cytometry. Bars represent the total live cell count as a fraction of ns-siRNA control (± s.e.m.). Asterisks indicate significance relative to ns-siRNA controls **$p \leq 0.01$; $n = 3$). (**C**) MitoSOX™ assay of oxidative stress in MiaPaCa-2 grown for 48 h under normal cell culture conditions (20% O_2) or hypoxia (1% O_2).

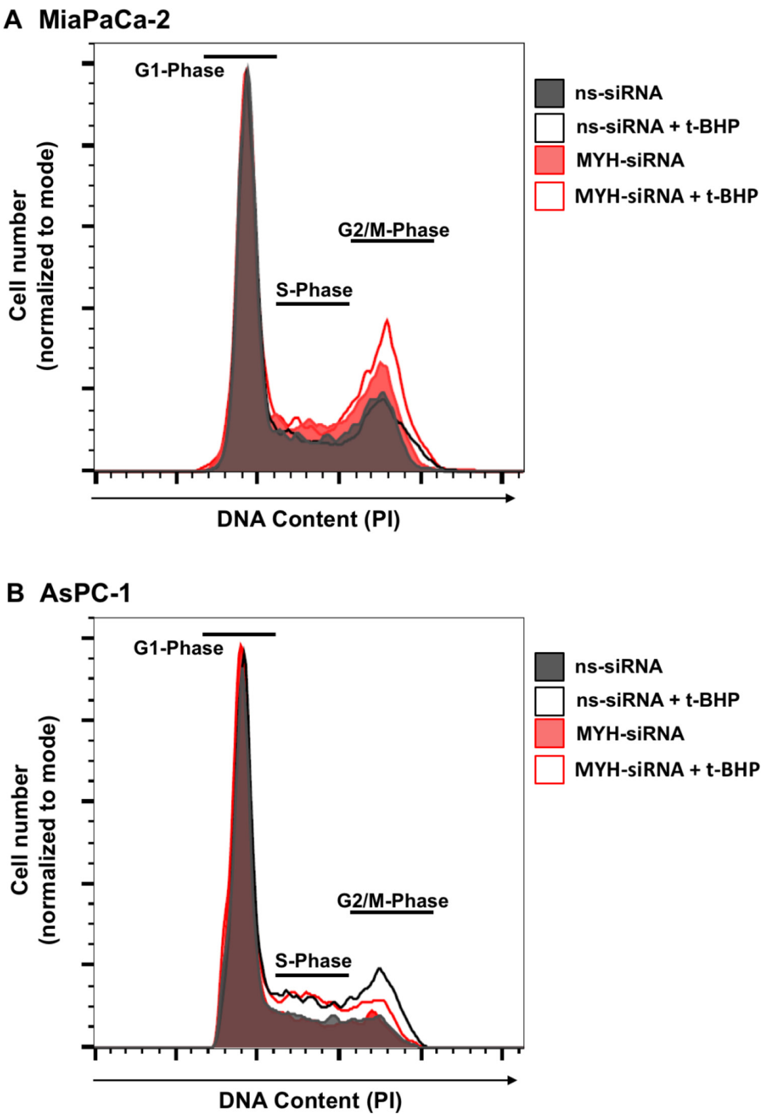

Supplementary Figure S3: The effect of MYH knockdown cell cycle in pancreatic cancer cells. (**A**–**B**) Flow cytometry plots of DNA content (propidium iodide, PI). MiaPaCa-2 and AsPC-1 cells were transfected with ns-siRNA or MYH-siRNA. 48 h post-transfection, cells were cultured in t-butyl hydroperoxide (t-BHP; 74 µM for MiaPaCa-2; 148 µM for AsPC-1). 48 h later, cells were stained with PI and DNA content measured by flow cytometry.
